# Radiofrequency versus Cryoballoon Ablation of Atrial Fibrillation: An Evaluation Using ECG, Holter Monitoring, and Implantable Loop Recorders to Monitor Absolute and Clinical Effectiveness

**DOI:** 10.1155/2018/3629384

**Published:** 2018-03-12

**Authors:** Karapet Davtyan, Victoria Shatakhtsyan, Hermine Poghosyan, Alexandr Deev, Alexey Tarasov, Maria Kharlap, Svetlana Serdyuk, Georgy Simonyan, Sergey Boytcov

**Affiliations:** ^1^National Research Center for Preventive Medicine, Moscow, Russia; ^2^Astghik Medical Center, Yerevan, Armenia

## Abstract

**Introduction:**

While several studies have compared the radiofrequency current (RFC) and cryoablation for the treatment of patients with atrial fibrillation (AF), no study has monitored the long-term outcomes with the usage of implantable loop recorders (ILRs).

**Methods:**

We enrolled 89 consecutive patients with nonvalvular paroxysmal AF (*N* = 44 for RFC and *N* = 45 for cryoballoon). The primary efficacy end point was the assessment of effectiveness for each group (RFC versus cryoballoon) when examining freedom from arrhythmia by monitoring with ECG, Holter, and implantable loop recoder (ILR). The primary safety end point compared rates of adverse events between both groups. The secondary efficacy end point examined the duration of the postablation blanking period from ILR retrieved data.

**Results:**

The mean age of the study population was 56.6 ± 10.2 years, and the follow-up duration was 12 months. There were no differences in baseline patient characteristics between groups. At 12 months, the absolute effectiveness (measured by ILR) was 65.9% in the RFC group and 51.1% in the cryoballoon group (OR = 1.85; 95% CI: 0.79–4.35; *p* = 0.157), and the clinical effectiveness (measured by ECG and Holter) was 81.8% in the RFC group and 55.6% in the cryoballoon group (OR = 3.6; 95% CI: 1.37–9.46; *p* = 0.008). There was no difference in safety between both groups. Asymptomatic episodes were significantly more present in the RFC group as measured by ILRs (*p* < 0.010). In cryoballoon group, arrhythmia episodes were recorded equally irrespective of the follow-up method (i.e., ECG and Holter versus ILR (*p* > 0.010)). The blanking period does not seem to be as important in cryoballoon as compared to RFC.

**Conclusion:**

RFC and cryoballoon ablation had similar absolute effectiveness at 12 months. ECG and Holter were effective when assessing the efficacy of the cryoballoon ablation; however, in the RFC group, ILR was necessary to accurately assess long-term efficacy.

## 1. Introduction

Pulmonary vein isolation (PVI) by catheter ablation is now a cornerstone treatment strategy for patients with symptomatic and drug refractory atrial fibrillation (AF) [1–3]. The 2016 ESC guidelines recommend that catheter ablation by PVI should be considered as a first-line treatment strategy for patients with AF and that the radiofrequency current (RFC) and cryoballoon ablation catheters are both effective for PVI [4]. Recently, the FIRE AND ICE trial reported both primary and secondary study results [5, 6]. The time-to-first event analysis for both safety and efficacy demonstrated equivalency between the two ablation catheters (RFC versus cryoballoon); however, the secondary end points examined continued reinterventions and rehospitalizations beyond the primary efficacy index failure event. In the FIRE AND ICE trial, there were significant reductions in the cryoballoon treated group (compared to the RFC cohort) when examining these additional study end points, mainly: all-cause rehospitalizations were reduced by 21%; cardiovascular rehospitalizations were reduced by 34%; repeat ablations were reduced by 33%; and direct current cardioversions were reduced by 50%. The study authors reported that these clinical differences may have been the result of a differential clinical arrhythmia burden between the two catheters that occurs after the primary efficacy end point reporting event, but they candidly stated that implantable loop recorders (ILRs) were not used in the FIRE AND ICE study to fully assess the impact of arrhythmia burden on these patients. In our current study, the investigators compared the RFC and cryoballoon catheters in patients with AF. The primary ablation strategy was PVI, and patients were monitored by ILRs and conventional methods (ECGs and Holter recordings). To our knowledge, this is amongst the first studies to report a RFC versus cryoballoon long-term result while examining both symptomatic and asymptomatic AF with the usage of ILRs.

## 2. Methods

### 2.1. Study Design and Population

The primary end point of this study was the 12-month assessment of the freedom from atrial arrhythmia occurrence when comparing the effectiveness of RFC versus cryoballoon catheter ablation between the two groups of treated subjects. In both groups, the primary ablation strategy was PVI and additional linear lesions were only used to create a cavotricuspid line of block in patients with confirmed typical right atrial flutter. Both acute procedural success and freedom from atrial arrhythmias were evaluated in the 12-month effectiveness end point. In this study, freedom from atrial arrhythmia was denoted by the lack of detection of AF (≥30 seconds in duration), atrial flutter, or atrial tachycardia episodes. Arrhythmia assessments were conducted by ECG, 24-hour Holter monitoring, and ILR. When atrial arrhythmias were detected by ECG and Holter, these episodes were denoted as “clinical effectiveness” end points; and when atrial arrhythmias were found by ILR reports, these episodes of arrhythmia were denoted as “absolute effectiveness” end points. The secondary end point was the evaluation of (the postablation) 90-day blanking period to determine the duration period needed by each catheter type to achieve long-term stable normal sinus rhythm after the healing of injuries associated with the cardiac ablation lesion formation as determined by ILR examination.

This study examined subjects with paroxysmal AF which was defined as ≥2 AF episodes that terminated spontaneously within 7 days, and it included subjects with AF episodes ≤ 48 hours that were terminated by electrical or pharmacological cardioversion. Patient eligibility into the trial was determined by inclusion and exclusion criteria. For inclusion into the trial, the patients met all of the following criteria: (1) ≥1 documented ECG occurrence of nonvalvular symptomatic paroxysmal AF lasting > 30 seconds within 90 days of enrollment that was refractory or intolerant to ≥1 antiarrhythmic drug (including beta blockers); (2) age ≥ 18 and ≤79 years; (3) left atrial diameter < 50 mm (anteroposterior) by parasternal long axis view; and (4) left ventricular ejection fraction ≥ 50% during sinus rhythm (estimated by Simpson's method). Patients were excluded from the trial if any of the following criteria was present: (1) a patient history of myocardial infarction or cardiac surgery within 90 days of enrollment; (2) a patient history of stroke or transient ischemic attack within 1 year of enrollment; (3) any uncontrolled thyroid dysfunction; or (4) a patient who was contraindicated or had an inability to maintain anticoagulation via oral pharmaceutical drug.

This trial was a prospective single center randomized study conducted with 89 consecutive subjects with nonvascular paroxysmal AF. The trial was funded by the Russian Federation which predefined the study timeframe and number of patients.

**Figure 1:**
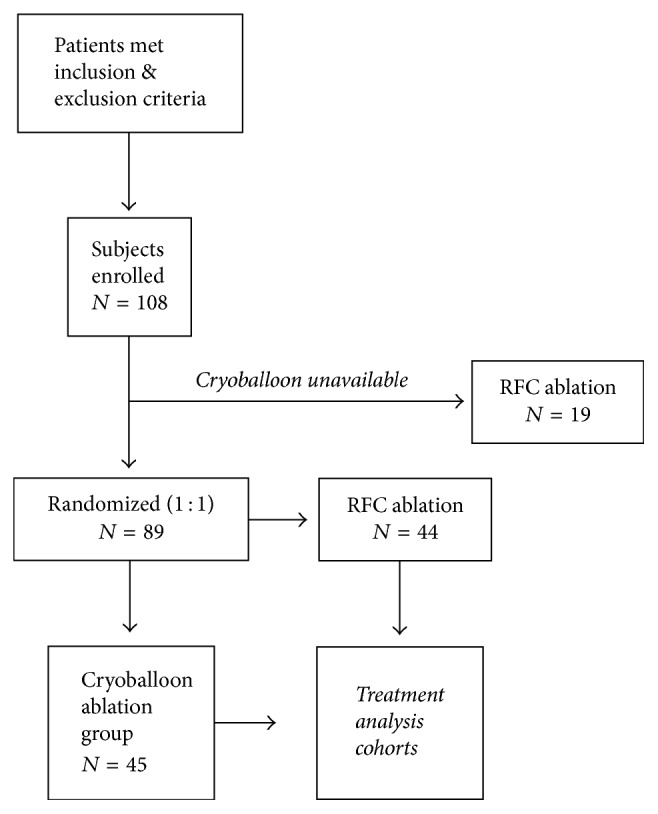
Patient disposition chart.

108 subjects were initially included in the study. Due to technical issues with the product supply chain, the first 19 subjects that met inclusion and exclusion criteria were treated with the RFC (Figure 1). These 19 subjects were not included in the statistical analysis. As a result, this trial evaluated 44 subjects treated with RFC catheter ablation and compared the outcomes to 45 subjects treated with a cryoballoon catheter. The patient enrollment started in March 2014, but the cryoballoon catheters were unavailable (for this study) until August 2014. The final subject enrollment occurred in July 2015, and the data set was locked in September 2016 after all subject follow-ups had been conducted. All subjects had given written informed consent before inclusion into the study, and the institutional review board approval of the study protocol was granted by the local ethics committee at the National Research Center for Preventive Medicine (Moscow, Russia).

### 2.2. Study Protocol and Procedures

Before the ablation procedure, all patients underwent a complete clinical history, including medical history review, physical examination, laboratory studies (including thyroid function testing), echocardiography, in-office ECG, and preablation Holter monitoring. All subjects were required to maintain an anticoagulation regime before the ablation procedure. If the subject was on warfarin, the international normalized ratio target was between 2 and 3 which was confirmed on the day before the ablation procedure. If the patient was taking a novel oral anticoagulant drug, the subject was required to maintain the pharmaceutical therapy for at least 4 consecutive weeks before the ablation procedure. All oral anticoagulant drugs were discontinued on the day before the procedure, and subjects were bridged with low-molecular weight heparin to maintain anticoagulation. On the day of the ablation procedure, a transesophageal echocardiography was performed to assess the left atrium for the presence of thrombi.

During the ablation procedure, subjects were sedated using general anesthesia which was initiated using propofol (2 mg/kg) and fentanyl (1-2 mg/kg). Venous access was obtained using a modified Seldinger technique, and two femoral venous routes were utilized. In the left femoral route, an 11 Fr sheath was utilized to deliver a 10 Fr phased-array ultrasound catheter (AcuNav, Acuson) which was used in the right atrium to visualize and direct the transseptal puncture. After transseptal puncture, the ultrasound catheter was removed, and the 11 Fr sheath was used to deliver a decapolar diagnostic catheter into the coronary sinus. In the right femoral route, an 8.5 Fr SL0 sheath was used to deliver the Brockenbrough needle (BRK, St. Jude Medical) for transseptal puncture. Immediately after transseptal puncture, a bolus of unfractionated heparin (100 U/kg) was administered, and an activated clotting time of ≥300 seconds was maintained throughout the ablation procedure with periodic heparin administration. Before catheter ablation, high-rate ventricular pacing was used to facilitate a left atriography.

Throughout the RFC catheter ablation procedure, a circular mapping catheter (LASSO, Biosense Webster) was positioned at the level of each pulmonary vein (PV) before each ablation. A 3.5 mm irrigated tip RFC ablation catheter (ThermoCool, Biosense Webster) was used, and RFC energy was delivered with a maximum temperature setting of 44°C and a power of 35 watts (with a flow rate of 17 ml/min of saline at the irrigated tip). RFC ablation catheter and multielectrode circular diagnostic catheter placement was facilitated with the usage of a 3D electroanatomical mapping system (CARTO XP, Biosense Webster).

During the cryoballoon ablation procedure, the cryoballoon was delivered to the left atrium over a guidewire using a dedicated cryoballoon catheter sheath (FlexCath, Medtronic). The 28 mm first-generation cryoballoon (Arctic Front, Medtronic) was used exclusively, and before each cryoablation, balloon-to-PV occlusion was tested with the injection of a radiopaque contrast agent. Confirmation of occlusion was demonstrated by the retrograde retention of contrast agent as viewed by fluoroscopy. At each PV, a 300-second cryoablation was performed, and 2-3 applications of cryoablation were used at each vein. During right-sided PV ablations, a decapolar diagnostic catheter was placed in the superior vena cava and cranial to the right superior PV in order to pace the right phrenic nerve (25 mA at a cycle length of 2 seconds) resulting in diaphragmatic contractions. Cryoablation was immediately terminated at any sign of diminished diaphragmatic contraction, and the cryoballoon was repositioned before the continuation of ablation. For all RFC and cryoballoon ablation procedures, acute PVI was tested by entrance and exit block testing, and cavotricuspid lesions were confirmed by line of block testing.

After each ablation procedure, an ILR (Reveal XT, Medtronic) was implanted in the subdermal space of the left superior chest of each subject, and AF detection parameters were set to evaluate R-R interval variability. Before discharge from the hospital, all subjects were evaluated by neurological examination, ECG, echocardiography, and visual assessment (of the femoral venous puncture site(s) for observation of bleeding). All complications were noted in the subject's medical records, and procedure related complications were further denoted as a study significant complication. At the time of hospital discharge, subjects were restarted on their previous oral anticoagulation drug and additionally given beta blockers. In cases of early recurrence of atrial arrhythmia during the 90-day blanking period, antiarrhythmic drugs (classes I and III) were used to manage the short-term arrhythmia symptoms with the exception of amiodarone which was not used in this study.

Study follow-up included in-office subject visits at 1, 2, 3, 6, and 12 months after the index ablation. During each visit, an ECG was recorded, and additionally, data were retrieved from the 24-hour Holter monitors and the ILRs. If subjects had a suspected arrhythmia event at any time during the 12-month follow-up period, additional in-office visits were completed to assess the potential arrhythmia using ECG, Holter monitoring, and ILR derived data.

### 2.3. Statistical Methods

Statistical data analyses were conducted using IBM SPSS Statistics 23. Mean values were given with a corresponding standard deviation, and discrete count data were accompanied by percentages. Continuous variables were analyzed using the *t*-test, and discrete variables were tested using Fisher's exact test. The Pearson's chi-squared test or Fisher exact test was used for nominal variables. Time-to-first recurrence of atrial arrhythmias was analyzed using Kaplan-Meier estimates, and overall freedom from atrial arrhythmias was compared between the 2 groups using the Mantel-Cox log-rank testing. Statistical significance was achieved at *p* < 0.05.

## 3. Results

The mean age of the study population was 56.6 ± 10.2 years, and the duration of subject follow-up was 12 months. Clinical patient demographic characteristics are reported in Table 1, and there was no statistical difference between the RFC and cryoballoon cohorts. Acute procedural success rates were similar between the two groups (RFC = 100% and cryoballoon = 97.8%; *p* = 0.240). In the RFC group, the mean procedure time was 152.4 ± 49.7 minutes while the mean procedure time in cryoballoon group was 147.7 ± 33.6 minutes which was statistically different (*p* = 0.013). The mean fluoroscopy time during the RFC procedures was 47.1 ± 18.8 minutes and 23.7 ± 12.5 minutes in the cryoballoon group which was statistically different (*p* < 0.001). Typical right atrial flutter ablation by cavotricuspid linear lesions was completed in 29.5% of the RFC group and 38% of the cryoballoon group which was not statistically significant (*p* = 0.503). The rates of procedural complications were comparable between groups (RFC = 4.5% and cryoballoon = 4.4%; *p* = 1.000). There were no periprocedural deaths, major bleeding events, or thromboembolic events during the study. In the cryoballoon group, transient phrenic nerve palsy was detected in 2 subjects (4.4%); however, both phrenic nerve dysfunctions resolved before the end of the ablation procedure. There was no phrenic nerve palsy at the time of hospital discharge for all subjects. In the RFC group, 2 subjects had an arteriovenous fistula (4.5%), and both patients were managed conservatively with monitoring and without further clinical intervention. In the RFC group 6 (13.6%) subjects and in the cryoballon group 13 (28.9%) subjects with symptomatic reccurences of the AF underwent RFC reablation (Table 2).

**Figure 2:**
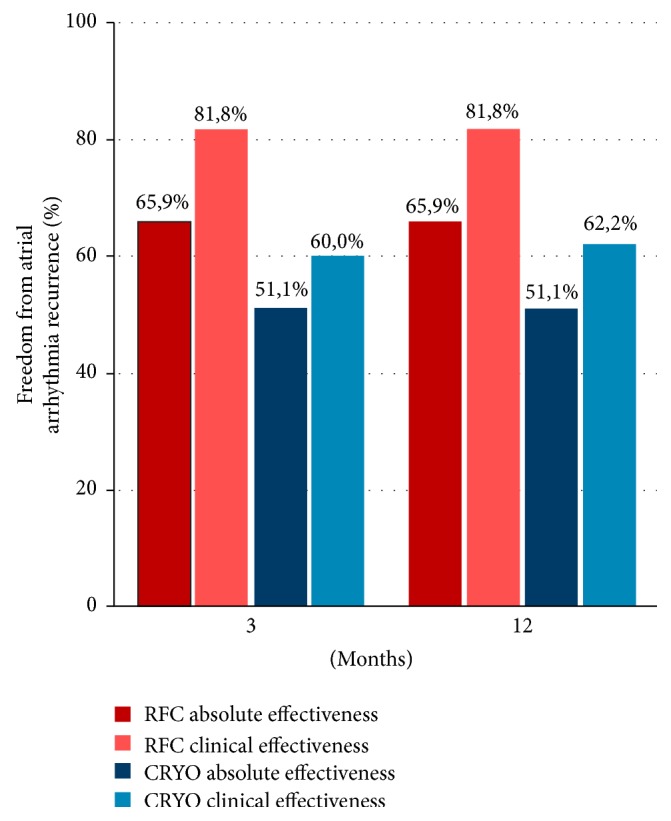
Effectiveness of ablation during the follow-up. Implantable loop recorders detected more episodes of arrhythmia recurrence compared to standard monitoring methods (ECG and Holter) after radiofrequency ablation. This difference was not significant after cryoballoon ablation.

**Figure 3:**
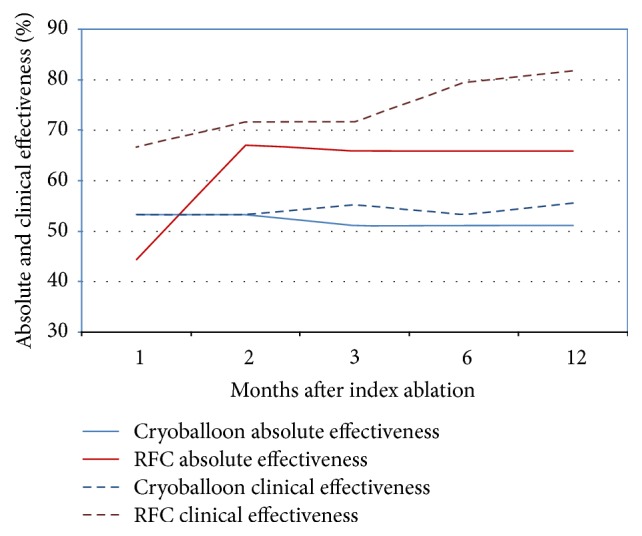
Arrhythmia recurrence rate during the 12-month follow-up.

There was 100% subject compliance at all scheduled follow-up visits (RFC = 220 visits and cryoballoon = 225 visits). Absolute effectiveness (as measured with ILR reporting) was 65.9% in the RFC group and 51.1% in the cryoballoon group at the end of the 12 month follow-up period. By comparison, clinical effectiveness as reported by ECG and 24-hour Holter monitoring was 81.8% in the RFC group and 55.6% in the cryoballoon treated group at the 12-month follow-up (Figure 2). There was a statistically significant improvement in atrial arrhythmia recurrence detection by ILRs for the RFC group (*p* < 0.001); however, there was no improvement by ILR detection for the cryoballoon group (*p* = 0.500) when compared to ECG and Holter monitoring. By analysis of variance testing, there was a significant increase in the atrial arrhythmia recurrence rate after the first month of follow-up for the RFC cohort (*p* < 0.001, Figure 3) and then there was a stable rate thereafter. However, there was no dispersion characteristic during the first month of follow-up found with the cryoballoon cohort, and the recurrence rate of atrial arrhythmias was statistically unchanged throughout the follow-up period (Figure 3). Total group analyses showed that 42% of ILR document atrial arrhythmias were completely asymptomatic; however, there were significantly more asymptomatic episodes in the RFC group (*p* = 0.010). By comparison, asymptomatic atrial arrhythmia episodes were recorded in the cryoballoon group irrespective of the follow-up method (EGG, Holter, or ILR).

## 4. Discussion

**Figure 4:**
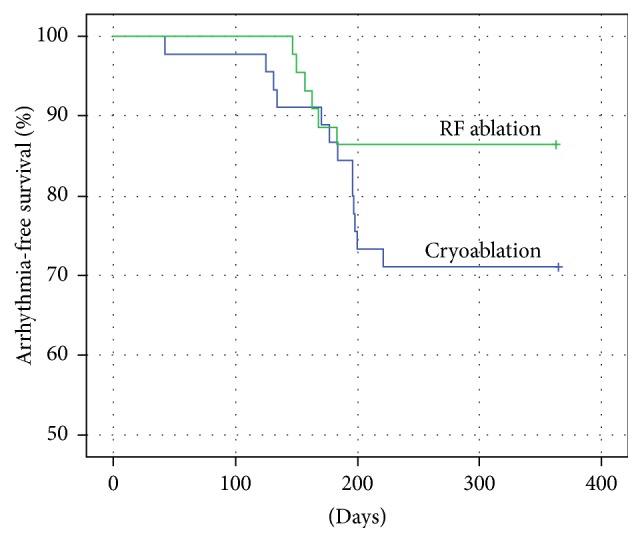
Kaplan-Meier estimates showing the cumulative freedom from all recurrent atrial arrhythmias after the radiofrequency ablation and cryoballoon pulmonary vein isolation. The average period of the recurrent arrhythmia development in the RFC group was 337,2 ± 10,6 days (95% CI: 316,5–357,9) and 307,8 (95% CI: 280,6–335,0) in the cryoballoon group. Freedom from all recurrent atrial arrhythmias did not differ between the 2 groups when compared by Mantel-Cox log-rank testing (*p* = 0.097).

In our current study, the RFC and cryoballoon catheters were compared in patients with paroxysmal AF for both efficacy and safety; however, our study is one of the first clinical reviews of both ablation catheters while patients were under ILR surveillance during the follow-up period. The study results demonstrated that both catheters have equivalent efficacy when examining patients by ILR interrogation and reporting the absolute effectiveness at 12 months after the index ablation (freedom from atrial arrhythmia) (Figure 4). A similar rate of safety events was reported for both RFC and cryoballoon procedures; however, the most frequent adverse event for RFC was arteriovenous fistula while phrenic nerve palsy was the most frequent adverse event for the cryoballoon catheter procedure. In both patient groups, the adverse events resolved without further clinical intervention. When examining RFC and cryoballoon response, the absolute effectiveness demonstrated that the RFC group did have a significant increase in asymptomatic atrial arrhythmia recurrence after the first month of follow-up. Also, between both catheter groups, it was evident by ILR detection that asymptomatic recurrence of AF is substantial and that a continuous monitoring of AF is warranted after any catheter ablation of AF, especially if the patient is changing or discontinuing anticoagulation therapy.

Recently, several RFC versus cryoballoon studies have been reported, and meta-analyses of these trials have demonstrated that RFC and cryoballoon share similar efficacy and safety profiles [7–10]. In fact, the FIRE AND ICE trial is the largest multicenter prospective randomized trial on catheter ablation, and this trial also demonstrated an equivalency between catheter groups with regard to safety and efficacy [5]. However, our study further examined both symptomatic and asymptomatic AF episodes after the index ablation by using continuous loop monitors. ILRs are more sensitive to AF detection compared to intermittent monitoring, especially for the detection of asymptomatic AF. The algorithm used for AF detection in the Reveal XT ILR has been shown to be 98.5% accurate [11], and it has been previously shown that 12% of patients had asymptomatic recurrences [12]. These asymptomatic episodes were shorter and slower and had lower heart rate variability [12]. However, until our study, limited data were available on asymptomatic AF recurrences after catheter ablation, and even a lesser amount of data is available after cryoballoon ablation.

Specifically, our study demonstrated the prevalence of the absolute effectiveness end points following a cryoballoon ablation (suggesting a prevalence of symptomatic AF recurrence following a cryoballoon ablation). In our study, there were two cryoballoon treated patients with asymptomatic recurrences: one patient had a short burst of AF at night time and the other patient had an AF episode with a heart rate that was near normal range. By comparison, RFC ablation had a lower incidence of the efficacy end point failure by clinical effectiveness examination, but the absolute effectiveness was matched with the cryoballoon reporting in our study. These findings demonstrated that standard monitoring methods (ECG and Holter) may overestimate the effectiveness of RFC ablation. Furthermore, a true comparison of RFC versus cryoballoon technology should be done in a large randomized population while using ILRs. Neither FIRE AND ICE nor the current meta-analyses use ILRs [5–10], and the true results of RFC efficacy could be overestimated while by comparison the cryoballoon results may be underestimated.

## 5. Limitations

This study was one of the first comparison trials conducted in the Russian Federation comparing RFC and cryoballoon ablation, and hence, the current sample size is small, clinical to other recent randomized studies. Also, the study was conducted on older technology, and it is known that both RFC and cryoballoon now have advanced catheter offerings.

## 6. Conclusion

In our study, RFC and cryoballoon ablation had similar absolute effectiveness at 12 months after the initial index ablation. Traditional follow-up methods (ECG and Holter) were effective when assessing the efficacy of the cryoballoon ablation because of the infrequent nature of asymptomatic episodes of atrial arrhythmias; however, in the RFC group, a continuous monitoring method (ILR) was necessary to accurately assess long-term efficacy. The effectiveness of cryoballoon ablation may be assessed within a few days after index ablation, as it remained stable during the one-year follow-up period suggesting that the cryoballoon procedure may not require a blanking period following the index ablation.

## Figures and Tables

**Table 1:** Patient demographic characteristics at baseline.

Patient characteristic	RFC (*N* = 44)	Cryoballoon (*N* = 45)	*p* value
Age (mean ± SD)	55.6 ± 12.0	57.6 ± 8.2	0.364
Age > 60 years, *n* (%)	16 (36.4%)	18 (40.0%)	0.828
Male, *n* (%)	19 (43.2%)	22 (48.9%)	0.672
BMI kg/m^2^ (mean ± SD)	29.8 ± 4.2	29.9 ± 4.0	0.981
LA diameter, cm (mean ± SD)	4.0 ± 0.4	4.1 ± 0.3	0.129

CHA2DS2-VASc score, *n* (%)			
Mean	1.3 ± 1.0	1.3 ± 0.8	0.971
0	11 (25.0%)	7 (15.6%)	0.379
1	14 (31.8%)	20 (44.4%)	
2	13 (29.5%)	15 (33.3%)	
3	6 (13.6%)	3 (6.7%)	
History of TIA, *n* (%)	4 (9.1%)	5 (11.1%)	1.000
IHD, *n* (%)	2 (4.5%)	4 (8.9%)	0.677
Hypertension, *n* (%)	34 (77.3%)	35 (77.8%)	1.000
Diabetic mellitus, *n* (%)	6 (13.6%)	2 (4.4%)	0.157

Drugs, *n* (%)			
Antiarrhythmic drugs	44 (100.0%)	45 (100.0%)	1.000
Anticoagulation	44 (100.0%)	45 (100.0%)	1.000

**Table 2:** Long-term ablation effectiveness.

End point	RFC (*N* = 44)	Cryoballoon (*N* = 45)	OR	95% CI	*p* value
Absolute effectiveness, *n* (%)	29 (65.9)	23 (51.1)	1.85	0.79–4.35	0.157
Clinical effectiveness, *n* (%)	36 (81.8)	25 (55.6)	3.6	1.37–9.46	0.008
Reablation, *n* (%)	6 (13.6)	13 (28.9)	0.39	0.13–1.14	0.12

## Abbreviations

AF: Atrial fibrillation
ILR: Implantable loop recorder
PV: Pulmonary vein
PVI: Pulmonary vein isolation
RFC: Radiofrequency current.
